# Structure and Properties of ZnO Coatings Obtained by Atomic Layer Deposition (ALD) Method on a Cr-Ni-Mo Steel Substrate Type

**DOI:** 10.3390/ma13194223

**Published:** 2020-09-23

**Authors:** Marcin Staszuk, Daniel Pakuła, Łukasz Reimann, Mariusz Król, Marcin Basiaga, Dominika Mysłek, Antonín Kříž

**Affiliations:** 1 Department of Engineering and Biomedical Materials, Faculty of Mechanical Engineering, Silesian University of Technology, Konarskiego 18A, 44-100 Gliwice, Poland; daniel.pakula@polsl.pl (D.P.); lukasz.reimann@polsl.pl (Ł.R.); mariusz.krol@polsl.pl (M.K.); 2 Department of Biomaterials and Medical Device Engineering, Faculty of Biomedical Engineering Silesian University of Technology, Gliwice, Roosevelta 40, 41-800 Zabrze, Poland; marcin.basiaga@polsl.pl; 3 Systemy Przetwarzania i Integracji Danych sp. z o.o., Jarosława Dąbrowskiego 9, 44-200 Rybnik, Poland; dominika.myslek@gmail.com; 4 Department of Materials and Metallurgy Engineering, Faculty of Mechanical Engineering, University of West Bohemia, Univerzitni 22, 30614 Plzen, Czech Republic; kriz@kmm.zcu.cz

**Keywords:** nanostructured, mechanical properties, atomic layer deposition, ALD, zinc oxide, ZnO, 316L stainless steel, corrosion resistance

## Abstract

This paper aimed to investigate the structure and physicochemical and tribological properties of ZnO coatings deposited by ALD on 316L stainless steel for biomedical applications. To obtain ZnO films, diethylzinc (DEZ) and water were used as ALD precursors. Zinc oxide layers were deposited at the same temperature of 200 °C using three types of ALD cycles: 500, 1000 and 1500. The structure and morphology of ZnO coatings were examined using SEM and AFM microscopes. The XRD and GIXRD methods were used for the phase analysis of the obtained coatings. To determine the resistance to pitting corrosion, potentiodynamic investigations and impedance spectroscopy were conducted in a Ringer solution at a temperature of 37 °C. The obtained results showed that the number of ALD cycles had a significant impact on the structure, morphology and corrosion resistance of the ZnO layers. It was found that after increasing the coating thickness of the ZnO on the material, its electrochemical properties determining the corrosion resistance also increased. Moreover, on the basis of the ball-on-plate tribological investigations, we found a significant reduction in the friction coefficient of the samples with the investigated coatings in relation to the noncoated substrates.

## 1. Introduction

Zinc oxide is a semiconductor, most often n-type, from groups II–IV. In the case of this oxide, the band gap energy (E_g_) is 3.3 eV at room temperature. The sp^3^ hybridized orbitals are created by s orbitals and p cations (Zn^++^), (O^− −^) anions, and then they bind and antibind. The binding energy of exocytone is 60 meV at room temperature; ZnO is widely used in electronics, optoelectronics and photovoltaics as a semiconductor layer due to these properties [1,2,3].

Zinc oxide has demonstrated protective, regenerative, slightly antibacterial and anti-inflammatory properties. It is characterized by the ability to block almost the entire spectrum of UV radiation. The subject of zinc oxide as a biomaterial is relatively new. Zinc oxide, due to its electrical, slightly antibacterial and protective properties, is a material suitable for use in modern biomedical engineering. The latest publications have focused on improving the piezoelectric properties and wettability of implants, and they relate to biomaterials with a ZnO layer. The influence of doping coatings with ZnO nanoparticles has also been investigated. Investigations confirmed that it works as a powerful biocide and antioxidant in both the biomedical and pharmaceutical industries. Besides, ZnO in the form of nanoparticles exhibits photocatalytic properties similar to those of titanium oxide, which, in combination with the lower cost of ZnO, may allow its use in large-scale water purification [4,5,6,7,8,9,10,11,12,13,14,15,16,17,18,19,20,21,22,23,24].

The surface properties of metal biomaterials have a significant impact on the initial phenomena occurring on the surface of the implemented material. Physicochemical reactivity is treated as the criterion for the suitability of a biomaterial in a physiological environment. After implantation of the biomaterial into the living organism, it comes into direct contact with body fluid. Dynamic phenomena occurring on the surface of the biomaterial, which take place after its implementation, will have a strong impact on the life expectancy of the implant [4,5,12,13,14,15,16,24,25].

Thin ZnO layers can be applied via several techniques, by: magnetron sputtering, sol-gel method, using a pulse laser deposition (PLD), vapor deposition with the MOCVD organometallic method, using a MBE epitaxial molecular beam and atomic layer deposition (ALD) [1]. Among the mentioned methods, the ALD technique in particular allows one to obtain very high-quality ZnO layers. The deposition of the atomic layer by the ALD method only proceeds through chemical reactions on the surface of the substrate, leading to a self-limiting layer-by-layer growth. During the reaction in the ALD process, pulses of precursor materials are introduced alternately into the reactor one after the other. After each precursor cycle, the reactor is purged with an inert gas such as nitrogen. The purpose of blowing is to remove all byproducts of chemical reactions, except those that are chemisorbed on the surface of the substrate. Ultimately, as a result of the sequential surface reaction, an ultra-thin layer is formed. The advantages of the ALD method include, among others: an accurate control of the thickness of the deposited layers, good reproducibility, high homogeneity over a large area, excellent compatibility, denseness and compactness without discontinuities or defects in the structure, and low growth temperatures (in the case of this layer, the temperature window is in the range of 100–200 °C) [2,26,27,28].

Considering, first of all, the biomedical properties of zinc oxide, it should be emphasized that there is a need to constantly assess the potential adverse health effects of metal nanoparticles and metal oxides, including ZnO, in order to ensure their safe use [29,30,31,32,33,34]. Particular attention should be paid to the assessment of the cytotoxicity of ZnO nanoparticles in the human body in short- or long-term exposures. Many authors in their papers [30,34] show that zinc oxide is characterized by a selective toxicity in the case of neoplastic cells. These investigations confirm that ZnO nanoparticles induce toxicity in a cell-specific and proliferation-dependent manner, with rapidly dividing cells being the most susceptible, and inactive cells being the least sensitive. The very large difference in the cytotoxic response between cancer cells and their normal counterparts, as well as the differences between activated and resting T lymphocytes, indicate the enormous potential of ZnO nanoparticles as a unique approach for cancer cell chemotherapy and radiotherapy, as well as in new methods for the treatment of autoimmunity [30]. In contrast, the authors in paper [32] concluded that further investigations were needed to elucidate the influence of the interaction of ZnO nanoparticles with biomass on potential toxicity and nutrient absorption after long-term exposure. ZnO has been shown to interact with human body fluids in terms of changes in physicochemical properties, solubility, fluorescence quenching and corona formation between molecules and plasma proteins. Meanwhile, [33] found that the presence of ZnO nanoparticles did not significantly affect toxicity in relation to the components of the circulatory system, with an emphasis on the interaction with palmitate (PA) or lipopolysaccharide (LPS), which may occur when nanoparticles enter the blood.

In the case of the ZnO layer, the ALD temperature window has been investigated by many authors, and there exists no doubt regarding this matter [2,4,7,8,9,18,19,20,21,35,36,37]. Unfortunately, there is little research work related to determining the optimal number of ALD cycles for this layer applied to a steel substrate, which would ensure the best performance properties, in particular corrosion resistance. Moreover, experience from investigating other types of coatings, including TiO_2_, shows that the corrosion resistance may be inversely proportional to the number of ALD cycles [26]. Therefore, the purpose of this work was to investigate the impact of the number of ZnO coating deposition cycles by the ALD method applied on a Cr-Ni-Mo austenitic steel substrate on the structure, morphology and corrosion resistance of the obtained layers. The tribological properties of the obtained zinc oxide layers were also investigated.

## 2. Materials and Methods

**Figure 1:**
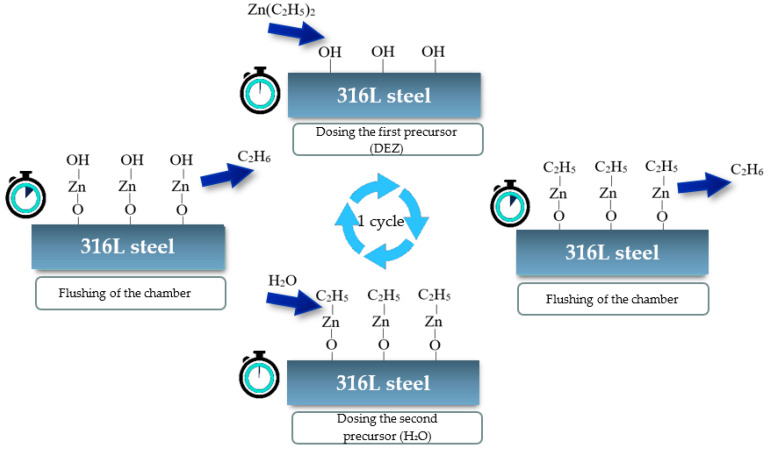
Stages of ZnO layers’ formation in the ALD process.

As a substrate material, flat cylindrical samples of steel grade 1.4404/316L were used. Samples were mechanically polished on SiC papers with 220, 500, 800, 1200 and 4000 grit and on a diamond suspension of 1-µm diameter. After that, the samples were thoroughly cleaned in acetone and then dried. ZnO zinc oxide layers were deposited with the use of the ALD (atomic layer deposition) process. The temperature was set at 200 °C, which is located in the so-called “growth window”. Diethylzinc (DEZ) and water were used as zinc and oxygen precursors, while nitrogen was used as the inert gas. Zinc oxide layers were deposited using three types of ALD cycles: 500, 1000 and 1500. For the sake of comparison, one sample remained uncoated. One cycle of the ALD process is presented in the diagram in Figure 1.

The structure and morphology of the surface were observed using a high-resolution scanning electron microscope (SEM) SUPRA 35 by Zeiss (Oberkochen, Germany) with the use of In Lens detection. Accelerating voltage ranging from 3 to 5 kV and the observations were carried out at a magnification of 150–200 kx. Additionally, for the changes resulting from the performed electrochemical investigations, an SEM microscope was used. Brittle fractures were prepared for observation by notching the substrate side notches on the samples, which were coated by the test coatings and cooled in liquid nitrogen before breaking.

Chemical composition analysis was carried out using EDAX X-ray energy dispersive spectroscopy (Mahwah, NJ, USA). The study was performed on the basis of images obtained from the SEM microscope using secondary electrons (SE) detection, 15 ÷ 20 kV acceleration voltage, and 50 kx magnification.

The morphology of the surface was examined by means of an AFM XE-100 atomic force microscope from Park Systems (Suwon, Korea). The study used the noncontact mode, and the scanned area was 1 μm × 1 μm.

The analyses of the phase composition of the substrates and of the obtained coatings were carried out using the X-ray diffraction method (XRD) on the X-ray apparatus X’Pert of the Panalytical (EA Almelo, The Netherlands), using the filtered radiation of a cobalt lamp.

To evaluate the electrochemical properties of samples with a zinc oxide layer and an uncoated sample, potentiodynamic investigations were carried out in a Ringer solution at a temperature of 37 °C, in order to provide the most accurate representation of the conditions prevailing in the human body. An ATLAS-SOLLICH ATLAS 0531 EU potentiometer (Rębiechowo, Poland) with a three-electrode measuring system was used in the study. A silver chloride electrode (Ag/AgCl) was used as the reference electrode, the platinum electrode was the supporting electrode and the anode was the investigated sample. At first, the open circuit potential was established at electroless conditions, after which the potentiodynamic polarization curves where registered. On the basis of the study, the following parameters were determined: Open circuit potential (E_ocp_), corrosive potential (E_corr_), breakdown potential (E_b_), repassivation potential (E_cp_), polarization resistance (R_p_) and corrosive current density (i_corr_).

An electrochemical examination by impedance spectroscopy (EIS) was performed due to the possibility to determine the characteristics and quality of the produced layers. The method was based on examining the system response at a determined free potential and a set frequency, in the form of a current pulse with an amplitude reduced by the resistance of the sample and the phase shift relative to the excitation pulse. The frequency range used in the study varied from 100 kHz to 10 mHz. The results of the impedance measurements were presented in the form of Nyquist plots, i.e., a real-imaginary impedance curve [f (Re (Z) = −Im (Z))] and Bode plots, i.e., two curves representing the frequency dependence on the impedance and phase shift [f (log (f) = log (Z) if (log (f)) = log (φ)]. The investigation was performed at a 10 mV amplitude.

The abrasion resistance studies were carried out using the Ball-on-plate method, using an Al_2_O_3_ ceramic ball with a diameter of 6 mm as a counter-sample. The research was carried out dry at room temperature. The counter-sample load was Fn = 1 N, with a friction path L = 200 m, linear speed v = 5 cm/s and wear radius r = 4 mm. The wear trace width was measured using a stereoscopic microscope. Observations of the resulting damage were made in an SEM microscope.

## 3. Results and Discussion

### 3.1. Morphology and Structure

**Figure 2:**
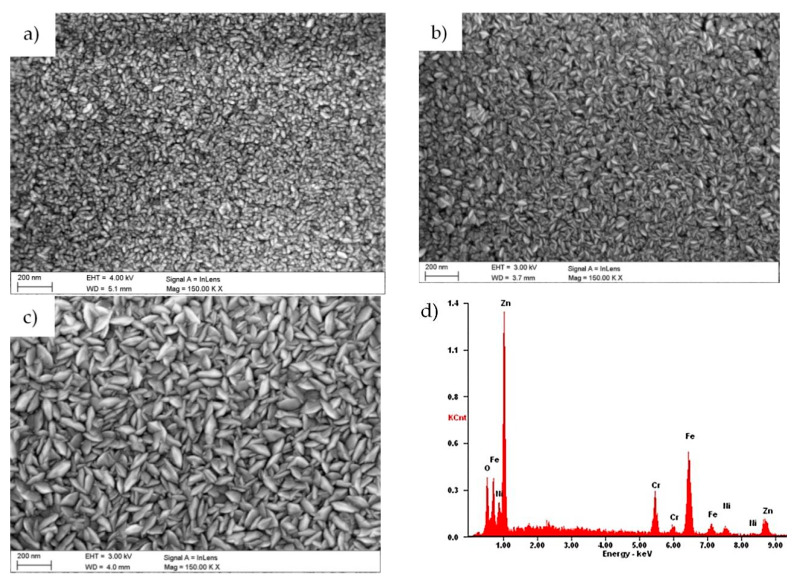
Surface morphology of the sample with the ZnO coating deposited in (a) 500; (b) 1000; and (c) 1500 ALD cycles; (**d**) X-ray energy dispersive plot of the area shown in Figure 2c.

The observation with the use of SEM (Figure 2) revealed that the obtained coating was conformal and homogeneous and covered the entire surface of the investigated samples. The morphology of the ZnO layers did not show any discontinuities, cracks, pores or defects.

The analysis showed that the grain size and structure changed with the increase of the number of ALD cycles. The sample with the layer deposited in 500 cycles had spherical grains with a symmetrical shape (Figure 2a). In the case of samples with the ZnO layer deposited in 1000 and 1500 cycles, the grains had the shape elongated in one direction and narrowed in the other (Figure 2b,c). Grains were distributed randomly without a dominant direction.

The chemical composition of the investigated samples was confirmed by energy-dispersive X-ray spectroscopy. The analyses in the microareas are presented in Figure 2d, and they were performed using the SEM microscope. The presence of characteristic peaks of zinc (1.01 and 8.64 keV) and oxygen (0.52 keV) in all applied samples was confirmed by investigations on the chemical composition. Reflections from chromium, iron and nickel deriving from the substrate material were also recorded. On the basis of this study, it was also found that the reflections corresponding to Zn became more pronounced while the number of cycles of the ALD process increased.

**Figure 3:**
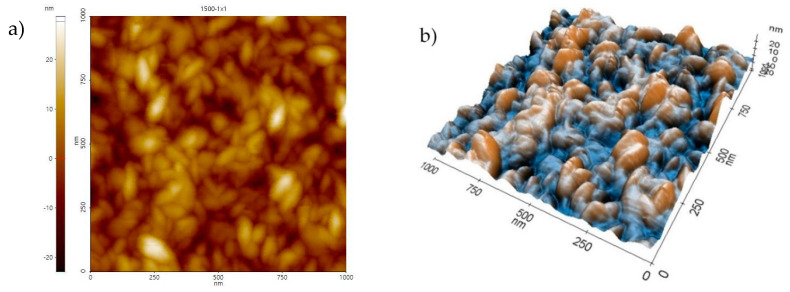
AFM scan of the surface morphology from a 1 × 1 μm^2^ area of samples with the ZnO coating deposited in 1500 ALD cycles; (**a**) 2D view, (**b**) 3D view.

**Figure 4:**
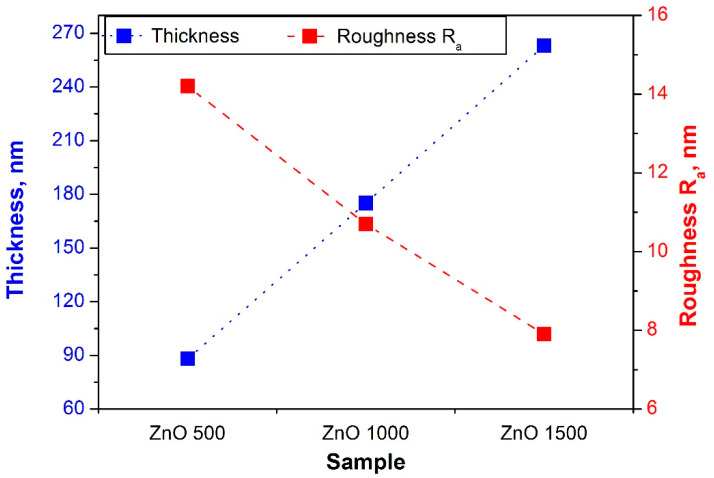
Thickness and roughness values of samples with a ZnO layer.

On the basis of the surface scans (Figure 3), it can be noticed that, as the number of ALD cycles increased, the deposited layer became more uniform. The average distance between the highest heights of the analyzed profile decreased, and their height assumed similar values. The presented regularity was reflected in the value of the R_a_ roughness parameter. As shown in Figure 4, the lower the number of ALD cycles was, the lower was the surface roughness. The surface quality increased significantly, as evidenced by decreasing the R_a_ parameter from 14 nm for the layer obtained at 500 cycles, to 7.8 nm for the coating applied at 1500 cycles.

**Figure 5:**
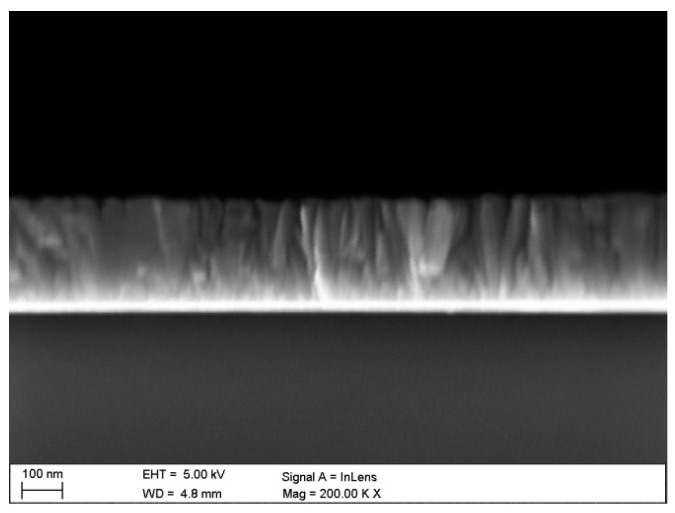
Fracture of the ZnO coating deposited in 1500 ALD cycles.

As a result of the observation of the fracture of the investigated coatings, it was found that the ZnO coatings showed a uniform structure without pores and discontinuities, and with a uniform thickness. Moreover, the investigated fractures did not show any delamination from the substrate (Figure 5). During the fracture investigations of the coatings, their thicknesses were measured and are presented in the diagram of Figure 4. The thickness of the investigated coatings ranged from 88 to 263 nm. It should be noted that the increase in the thickness of the coatings that depended on the number of cycles was linear in the investigated range, which meant that the rate of coating growth was constant and equalled about 0.18 nm/cycle.

**Figure 6:**
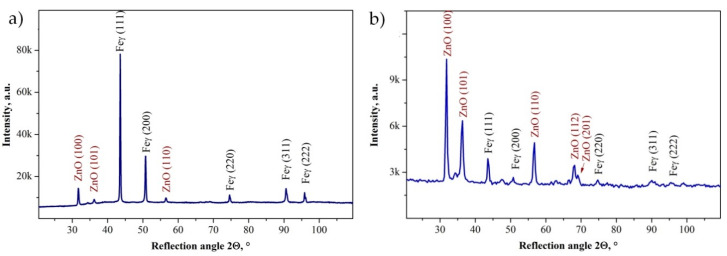
X-ray diffraction pattern of ZnO coating deposited on the Cr-Ni-Mo steel substrate obtained by the (**a**) Bragg-Brentano method and the (**b**) GIXRD method (α = 1°).

The qualitative analysis of the phase composition carried out with the X-ray diffraction method confirmed that the investigated coatings contained the crystalline ZnO phase (Figure 6). The presence of reflexes from the substrate was found on the diffraction patterns obtained by the Bragg–Brentano method, and was caused by the thickness of the obtained coatings, which was lower than the penetration depth of X-rays into the material. As a result of the investigations with the application of the grazing incident X-ray diffraction technique (GIXRD) at a low incidence angle (α = 1°) of the prime X-ray beam, we recorded the reflexes mainly from thin surface ZnO layers.

### 3.2. Electrochemical Properties

**Figure 7:**
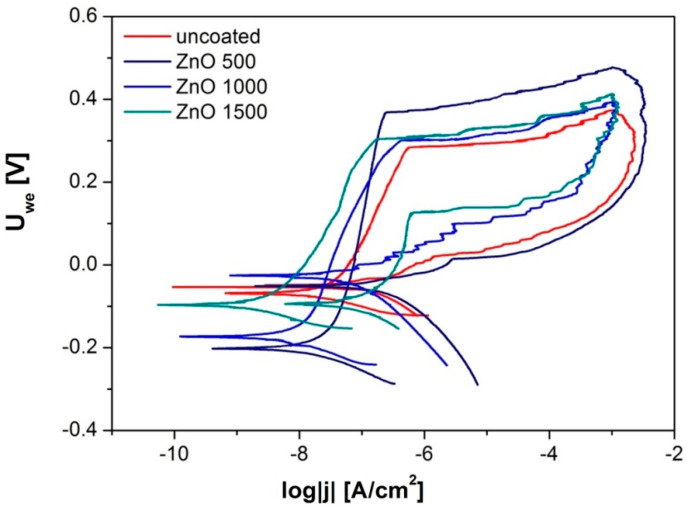
Anodic polarization curves for an uncoated sample and samples with ZnO layers.

The performed studies have proven the ability of all samples to reproduce the passive layer in an environment similar to that prevailing in the human body, as evidenced by the presence of the return curve. For all the registered curves, a hysteresis loop was obtained, indicating the initiation of pits and the course of corrosion. After the initiation of the pitting corrosion, the value of potentials decreased, and the process of rebuilding the passive layer began. Following the repassivation, the corrosion resistance of each investigated sample improved, which was proven by the shift of the potential value towards positive values (Figure 7).

By comparing the values of the density of the corrosion current (Table 1), one can note that the highest value was obtained for the uncoated steel. With the increase of the number of ALD cycles, the density of the corrosion current decreased. The opposite situation occurred in the case of the polarization resistance.

While the number of ALD cycles (i.e., the coating thickness) increased, the polarization resistance also increased from a value of 766 R_p_ kΩ∙cm^2^ (for the uncovered sample) to a value of 8317 kΩ∙cm^2^ (for the thickest coating).

In the recorded hysteresis curves (Figure 7), the shift in their characteristics visibly increased with the number of ZnO layer formation cycles on the steel substrate. The Tafel inflection pointed towards higher potential values than the potential of sample after 500 cycles by about 30 mV for the curve after 1000 cycles and by another 70 mV for the sample after 1500 cycles.

**Figure 8:**
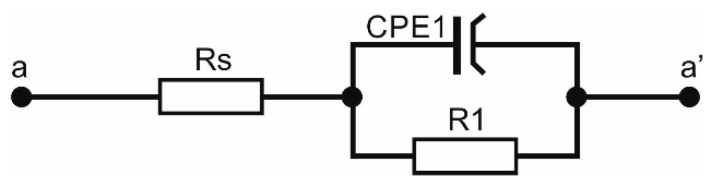
An equivalent circuit representing the impedance spectra of the investigated materials.

To match the equivalent electrical system to the obtained impedance spectra, the course of experimental curves was compared with the curves generated by the computer model. An equivalent circuit with a phase constant element (CPE-Constance phase element) and resistive elements was chosen, which reflected the resistance of the surface layer of the material and the electrical resistance of the tested electrolyte. The best match of electrical components to the obtained results was characterized by the circuit shown in Figure 8. The numerical values of the electrical model of equivalent circuit for the investigated materials are summarized in Table 2.

**Figure 9:**
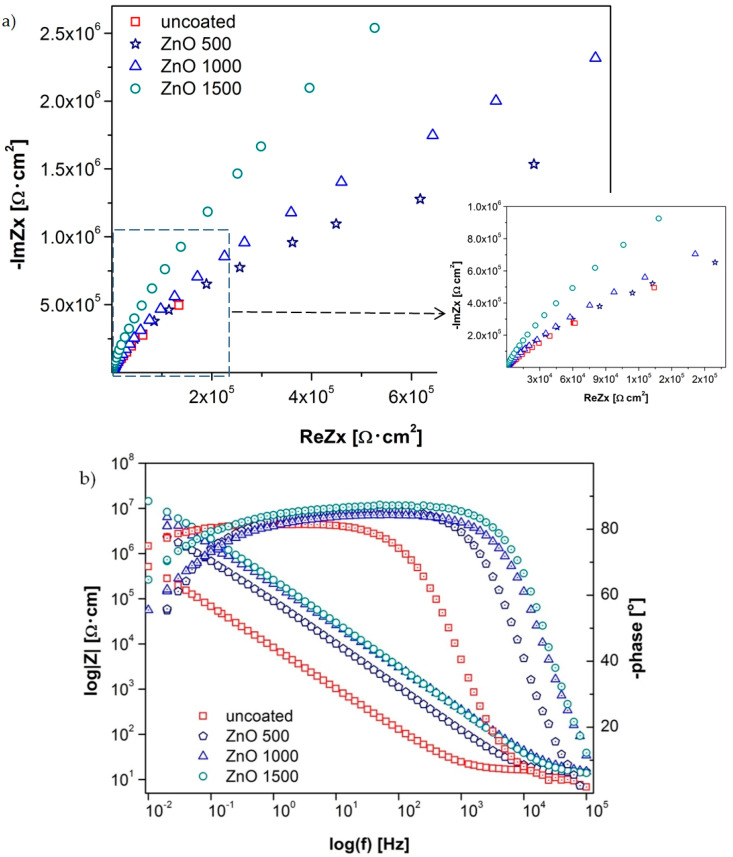
Impedance spectrum of the tested materials (for designated stationary potentials): (**a**) Nyquist representation and (**b**) Bode representation.

Based on the recorded data, by analyzing the course of the curves on the Nyquist chart, the impedance changes were visible depending on the number of cycles used to obtain the protective layer (Figure 9a). First of all, comparing the angle of inclination of the curves to the ordinate axis, it could be found that the most durable material in relation to corrosion damage was the material with the coating obtained in 1500 cycles, and the smaller the number of cycles was, the faster the corrosion damage increased.

Second, the impedance value of the material under test increased with the use of more cycles in the layer application, as evidenced by the increasing radius of the virtually drawn semicircle. Additionally, the shape of the recorded Nyquist curves, showing a course that was close to linear, could indicate the corrosion process, which was controlled by mass transport, i.e., the diffusion of cations from the corrosive metal. The analysis of the course of curves on the Bode chart indicated that the applied protective layers on the material contributed to the improvement of anticorrosive properties; in the entire range of frequencies investigated, the impedance of the substrate material was much lower than that of any sample with coatings.

Comparing the impedance values for the samples with the layers obtained for different numbers of cycles, it can be concluded that the greater the number of cycles, the greater the corrosion resistance, because the higher the impedance recorded at the investigated frequency range. The lowest impedance value for all investigated samples was, as is usually the case, recorded at high-frequency values of 10–100 kHz. The impedance value for the base material was 0.03–0.13 kΩ∙cm^2^ for average frequency values from 1000 to 100 Hz; for the layer after 500 cycles it changed from 0.12 to 1.11 kΩ∙cm^2^, for 1000 cycles it was 0.28–2.44 kΩ∙cm^2^, and for the layer after 1500 cycles it changed from 0.34 to 3.17 kΩ∙cm^2^. For low frequencies of 10–0.1 Hz, the differences in the impedance values were even more in favor of materials with coatings and amounted to 1–67 kΩ∙cm^2^ for the substrate sample, 10–679 kΩ∙cm^2^ for the material with a layer obtained in 500 cycles, from 21 to 1232 kΩ∙cm^2^ for the layer obtained after 1000 cycles, and from 29 to 2135 kΩ∙cm^2^ for the sample with a layer obtained in 1500 cycles (Figure 9b).

Both the good quality and stability, and the compact structure of the layer manufactured on the investigated materials were confirmed by the wide range of high phase shift angle values above 80° (Figure 9b), which was at a frequency of 20 to 0.06 Hz for the base material, in the range of 790–0.39 Hz for the sample with a layer obtained in 500 cycles, and from 1600 to 0.63 Hz for the material with a layer after 1000 cycles; furthermore, the widest frequency range, from 4000 to 0.15 Hz, was recorded for the sample with a layer obtained after 1500 cycles.

**Figure 10:**
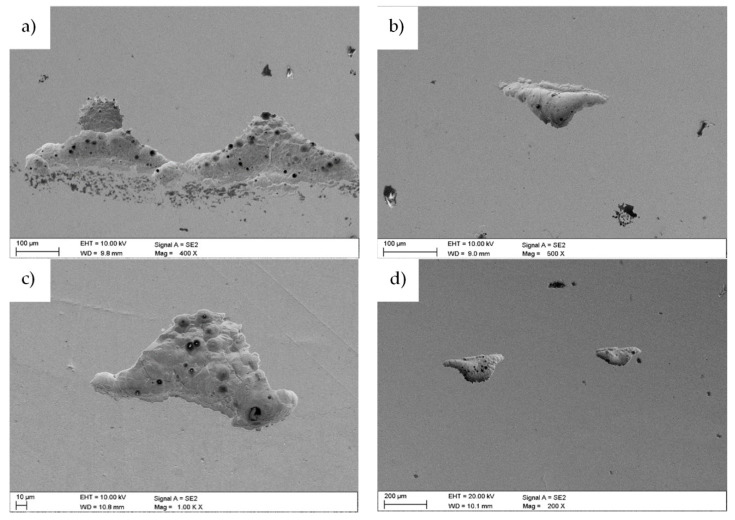
Surface morphology after an electrochemical examination of the sample: (**a**) uncoated; (**b**) with a ZnO coating deposited in 500 cycles; (**c**) 1000 cycles; and (**d**) 1500 cycles.

After the electrochemical investigations, the samples were subjected to microscopic observations to assess the corrosion changes. Figure 10 shows the results made by the use of SEM. During the electrochemical studies and investigations, pits were found in the applied samples. The pits had irregular shapes and sizes, and their structure consisted of etched grain boundaries of the base material. The largest number of pits were observed for the uncoated sample and the sample with the layer of zinc oxide deposited in 500 cycles (Figure 10a,b). The best resistance to pitting corrosion was displayed by the sample with a layer of ZnO deposited in 1500 cycles (Figure 10d).

**Figure 11:**
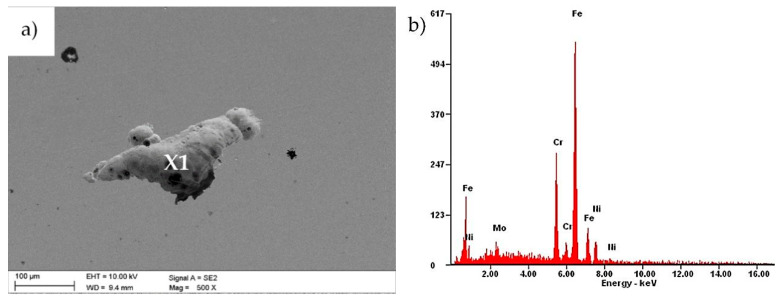
(**a**) Surface morphology after an electrochemical examination of an uncoated sample; (**b**) EDS spectrum from the microarea X1.

Parallel to this, a chemical composition analysis of selected areas was performed, using X-ray dispersion spectroscopy. The analysis for the uncoated sample in area X1 (Figure 11) showed the presence of elements such as iron, chromium, nickel and molybdenum, characteristic for the chemical composition of 316L steel. In the study, however, oxygen reflexes were not registered, which could confirm the presence of a passive layer formed on the sample surface. The reason may be the fact that the oxide layer was too thin to allow reflections from oxygen to be registered.

**Figure 12:**
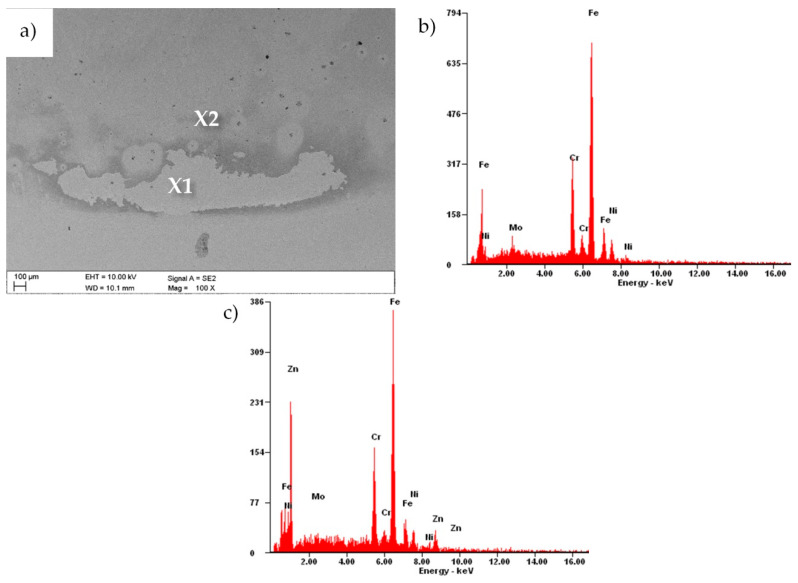
(**a**) Surface morphology after the electrochemical examination of a sample with a ZnO layer deposited in 1500 cycles; (**b**) EDS spectrum from the microarea X1 and (**c**) from the microarea X2.

The EDS results for the sample with the zinc oxide layer obtained in 1500 cycles revealed that the elements of the substrate material were chromium, nickel, molybdenum and iron (Figure 12b). In area X2, the presence of zinc was also recorded (Figure 12c), which indicated the delamination of the coating due to the electrochemical investigations.

### 3.3. Tribologycal Properties

**Figure 13:**
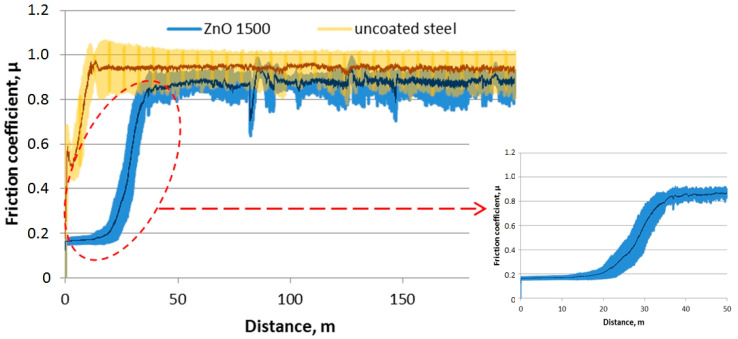
Friction coefficient as a function of the distance for the uncoated material and ZnO layer deposited in 1500 cycles.

**Figure 14:**
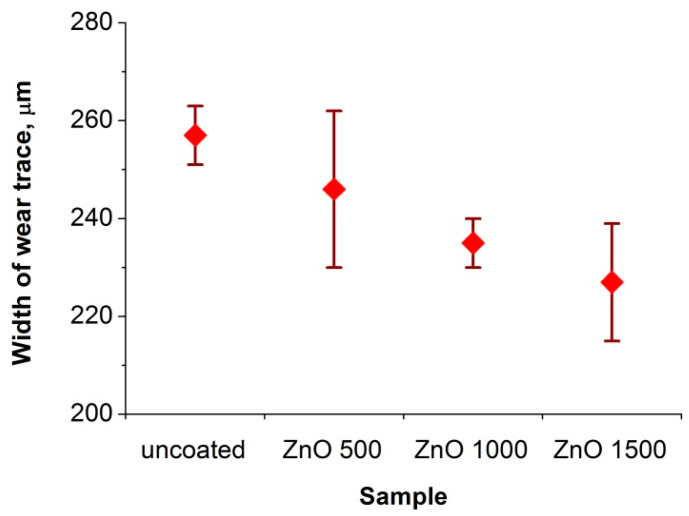
Width of wear trace of the investigated materials.

Figure 13 shows the chart for the friction coefficient (µ) as a function of the friction path for a sample made of both uncoated Cr-Ni-Mo steel and steel covered with a ZnO coating obtained in 1500 cycles. The friction coefficient for a sample coated with ZnO was initially valued at about 0.16, but there was a rapid increase in the value of this coefficient to about 0.85 after 22 m. This increase implied the abrasion of the ZnO layer onto the steel substrate. In the case of the uncoated sample, the friction coefficient increased rapidly from the initial value of 0.5 to the average value of 0.9, remaining at this level until the end of the investigation. In the case of the uncoated sample, the initial lower value of the friction coefficient was undoubtedly due to the presence of a passive layer on the austenitic steel surface, which was quickly removed, and the ceramic counter-sample rubbed against the steel substrate. Figure 14 presents the measurements of the width of wear. As the number of cycles of the ALD process increased, the average wear bandwidth decreased; however, the differences between individual samples were insignificant.

**Figure 15:**
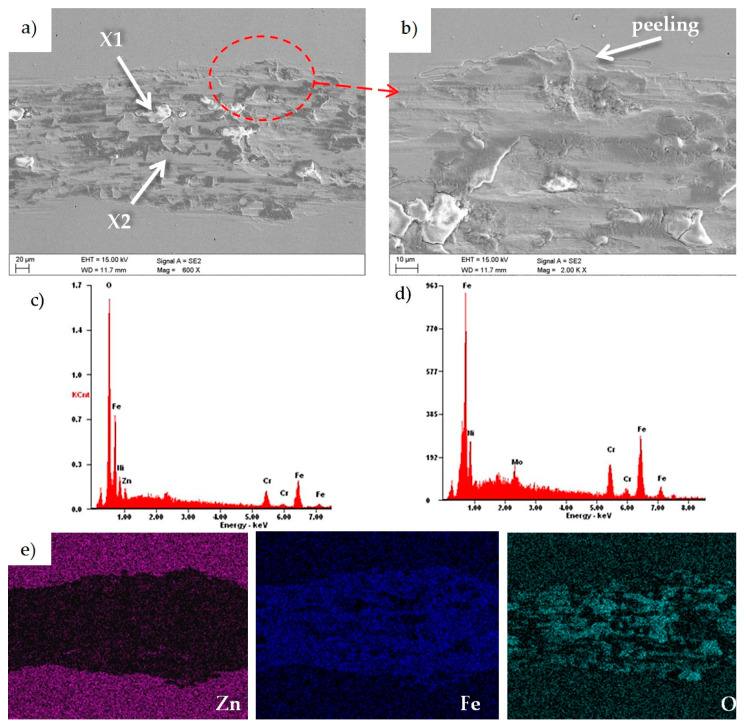
(**a**,**b**) Wear trace after the “ball-on-plate” wear test for the ZnO layer deposited in 1500 cycles, (**c**) X-ray energy dispersive plot of the area X1 shown in Figure 15a, (**d**) X-ray energy dispersive plot of the area X2 shown in figure, and (**b**,**e**) maps of superficial distribution chemical elements from the area shown in Figure 15a.

As a result of metallographic observations of the wear paths of the investigated samples (Figure 15) in the SEM, the presence of areas rich in oxygen and iron was found, which undoubtedly proved the formation of iron oxide Fe_2_O_3_. The main wear mechanism was abrasion, and at the edges of the wear path, few areas of peeling of the coating were found (Figure 15b).

**Figure 16:**
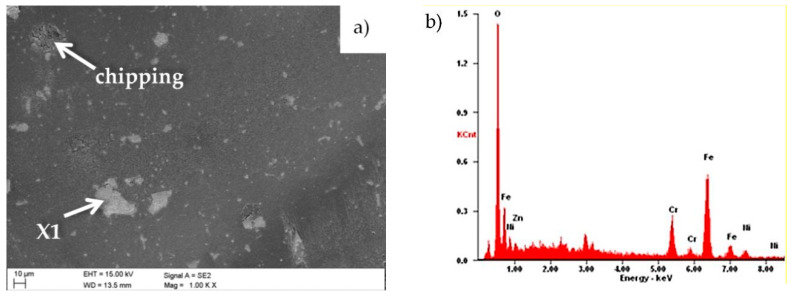
(**a**) Wear place after the “ball-on-plate” wear test for the Al_2_O_3_ ball as a counter-sample, (**b**) X-ray energy dispersive plot of the area X1 shown in Figure 16a.

Ceramic counter-samples were also investigated in SEM. The main wear mechanism of the counter sample was abrasion. Small areas with mainly oxygen and iron were also observed there, which proved the formation of iron oxide build-up. Small chippings (chipping) on the counter-sample-sample interface were also present (Figure 16).

## 4. Summary

Available literature sources provide information on the effect of the temperature or the time of precursor dosing on the structure and properties of zinc oxide coatings deposited in the ALD process. Nevertheless, these sources do not contain information on the effect of the number of cycles. This phenomenon has been examined and described in this paper. On the basis of the presented results, a correlation between the corrosion resistance and the roughness parameter was found, in which low values slowed the formation of corrosive cells. Besides, the corrosion resistance depends on the morphology of the surface, particularly on the shape and size of the grains. From a material engineering point of view, a combination of corrosion resistance and high surface quality enables a wide range of applications, especially in the field of implantology. Both parameters affect the final surface quality and constitute the main functional properties of the elements with zinc oxide coating.

Based on the performed research using the SEM microscope, homogeneous coatings without any pores or defects were obtained in the ALD process. It was found that the number of ALD cycles significantly affected the structure and morphology of ZnO layers deposited on 316L steel. With the increase in the number of cycles, the grains changed the size of the shape from spherical to more elongated. This proved that this phenomenon had a direct impact on the surface quality, which was confirmed during the study with the use of an atomic force microscope. Furthermore, the research showed a relationship between the number of cycles and the value of the roughness parameter R_a_ in a reciprocally proportional manner.

The chemical composition analysis of the obtained zinc oxide coatings confirmed that they consisted of zinc and oxygen, and a phase analysis confirmed the obtaining of the ZnO phase.

Electrochemical investigations demonstrated the ability of all tested samples to passivate. It follows that both the coated and reference samples showed resistance to pitting corrosion. Nevertheless, the biggest influence on the anticorrosion properties of the samples with the ZnO layer were the number of ALD cycles in which the coating was deposited. In the case of the investigated layers, the continuity of the ZnO coating was maintained, which almost completely protected the investigated samples from corrosion. Studies have shown that the increasing thickness of ZnO coatings, caused by the increasing number of ALD cycles, affected, among others, the increase of the polarization resistance, which translated into an increase in the corrosion resistance. This is the reverse tendency to, for example, the case of oxides of the TiO_2_ type obtained by the ALD method. The publication [26] shows that the optimal number of ALD cycles for TiO_2_ layers is 200, while layers obtained in a larger and smaller number of cycles show a lower corrosion resistance. It should be emphasized that the layers of TiO_2_ and ZnO, apart from differences in their chemical and phase composition, also differ in structure. While the titanium oxide layers show an amorphous structure, the investigated zinc oxide layers are crystalline. This is confirmed both by breakthrough microstructural studies using SEM microscopy, as well as by XRD and GIXRD diffraction studies. In turn, the crystallinity of zinc oxides obtained by this method is a characteristic feature, which was confirmed, among others, by the work of [2].

The investigation of corrosion changes using SEM microscopy confirmed the observed relationship between the number of cycles of the ALD process and the anticorrosion properties of samples with an ZnO coating. The smallest amount of pits and the best resistance to pitting corrosion were shown by the sample with the zinc oxide layer deposited in 1500 cycles.

The investigated ZnO coatings improved the tribological contact by reducing the coefficient of friction in relation to the uncoated sample. The obtained coefficient of friction of the investigated ZnO layers was approximately 0.16, which was a similar value to that obtained by Pietruszka et al. [21] by investigating zinc oxide on a glass substrate. In both cases, the counter-sample was an Al_2_O_3_ ceramic ball. The durability of the ZnO1500 coating in the tribological investigation was not very high. Moreover, the coating was rubbed to the substrate after 22 m of continuous friction. However, the durability of the coating was equal to 875 cycles under the given tribological conditions, but with a relatively small abrasive path diameter (8 mm).

The present investigations show that coatings can have a promising application in biomedical engineering, as well as for covering the surfaces of utility objects, which require a high corrosion resistance and biocidal properties characteristic for zinc oxide.

## 5. Conclusions

Based on the investigations, the following conclusions were drawn:

- The ZnO layer applied to the steel substrate improved the resistance to corrosion damage of the tested material, which was indicated by a decrease in the value of the corrosion current by one order of magnitude in relation to the uncoated sample and by an over 10-fold increase in the material resistance in the case of 1500 cycles. The increasing number of cycles by which the ZnO layer was obtained was conducive to obtaining better values for the electrochemical parameters of the investigated material, as evidenced by the increasing values of the corrosion potential and polarization resistance.
- The performed impedance spectroscopy tests confirmed the results obtained in the DC corrosion resistance tests, as indicated by the spectral characteristic of the impedance in the Nyquist plot and the increase of the curve slopes: there was an increase in the value of the impedance of materials with an increasing number of ZnO layer cycles, and an extension of the highest value of the phase shift angle in the Bode plot to an ever greater range of frequencies.
- As a result of the tribological investigations of the ZnO coatings, a slight increase in the abrasion resistance was found. Therefore, these coatings can be used in applications exposed to low abrasion. Moreover, our study may contribute to the further development of these coatings so as to increase their abrasion resistance.

## Figures and Tables

**Table 1:** Values obtained during potentiodynamic investigations.

Sample	E_corr_ (mV)	I_corr_ (µA/cm^2^)	R_p_ (kΩ∙cm^2^)
uncoated	−67.39	0.015	766.50
ZnO 500	−202.67	0.002	1486.95
ZnO 1000	−174.57	0.006	2752.55
ZnO 1500	−97.12	0.001	8317.38

**Table 2:** Values obtained during EIS investigations.

Sample	Rs (Ω∙cm^2^)	CPE1 (µF/cm^2^)	N1	R1 (kΩ∙cm^2^)
uncoated	0.01	0.12	0.001	586
ZnO 500	0.3	0.03	0.002	197
ZnO 1000	0.5	0.02	0.003	676
ZnO 1500	0.4	0.009	0.002	2350
